# Effect of Dietary Supplementation with Eufortyn^®^ Colesterolo Plus on Serum Lipids, Endothelial Reactivity, Indexes of Non-Alcoholic Fatty Liver Disease and Systemic Inflammation in Healthy Subjects with Polygenic Hypercholesterolemia: The ANEMONE Study

**DOI:** 10.3390/nu14102099

**Published:** 2022-05-18

**Authors:** Federica Fogacci, Elisabetta Rizzoli, Marina Giovannini, Marilisa Bove, Sergio D’Addato, Claudio Borghi, Arrigo F. G. Cicero

**Affiliations:** 1 Hypertension and Cardiovascular Risk Research Center, Medical and Surgical Sciences Department, Sant’Orsola-Malpighi University Hospital, 40138 Bologna, Italy; federicafogacci@gmail.com (F.F.); elisabetta.rizzoli@unibo.it (E.R.); marina.giovannini3@unibo.it (M.G.); marilisa.bove@aosp.bo.it (M.B.); sergio.daddato@unibo.it (S.D.); claudio.borghi@unibo.it (C.B.); 2 IRCCS Policlinico S. Orsola—Malpighi di Bologna, 40138 Bologna, Italy; 3 Italian Nutraceutical Society (SINut), 40138 Bologna, Italy

**Keywords:** dietary supplement, nutraceutical compound, cholesterol, artichoke, bergamot

## Abstract

We aimed to evaluate if dietary supplementation with a nutraceutical compound (Eufortyn^®^ Colesterolo Plus) containing standardized bergamot polyphenolic fraction phytosome (Vazguard^®^), artichoke extract (Pycrinil^®^), artichoke dry extract. (*Cynara scolymus* L.), Q10 phytosome(Ubiqosome^®^) and zinc, could positively affect serum lipids concentration, systemic inflammation and indexes of non-alcoholic fatty liver disease (NAFLD) in 60 healthy subjects with polygenic hypercholesterolemia. Participants were adhering to a low-fat, low-sodium Mediterranean diet for a month before being randomly allocated to 8-week treatment with 1 pill each day of either Eufortyn^®^ Colesterolo Plus or placebo. Dietary supplementation with Eufortyn^®^ Colesterolo Plus was associated with significant improvement in total cholesterol (TC), low-density lipoprotein cholesterol (LDL-C), non-high-density lipoprotein cholesterol (non-HDL-C), high-sensitivity C-reactive protein (hs-CRP) and endothelial reactivity (ER) in comparison with baseline, and with significant reductions in waist circumference, TC, LDL-C, LDL-C/HDL-C, lipid accumulation product and fatty liver index compared to placebo. The study shows that dietary supplementation with standardized bergamot polyphenolic fraction phytosome, artichoke extracts, Q10 phytosome and zinc safely exerts significant improvements in serum lipids, systemic inflammation, indexes of NAFLD and endothelial reactivity in healthy subjects with moderate hypercholesterolemia.

## 1. Introduction

Polyphenols are secondary plant metabolites and bioactive compounds naturally occurring in plants and plant-derived products [1].

Pooling data from several epidemiological and clinical studies, total flavonoids and specific subclasses have been associated with a reduced incidence of cardiovascular (CV) diseases (CVD), diabetes mellitus and all-cause mortality [2].

Flavonoids have been shown to act as free radical scavenging, and exert antioxidant, hepatoprotective and anti-inflammatory activities [3]. Actually, flavonoids’ biological activities reflect their chemical and biochemical properties, including the ability to regulate gene expression in chronic diseases and modulate several molecular pathways [4].

Recently, artichoke and bergamot standardized flavonoid extracts have been suggested as safe lipid-lowering nutraceuticals [5]. Based on this evidence, we aimed to evaluate if dietary supplementation with a nutraceutical compound containing standardized bergamot polyphenolic fraction phytosome^®^ and artichoke extracts could positively affect serum lipids concentration and, secondly, insulin sensitivity, systemic inflammation and indexes of non-alcoholic fatty liver disease (NAFLD) in healthy subjects with moderate hypercholesterolemia.

## 2. Materials and Methods

### 2.1. Study Design andParticipants

This was a randomized, double-blind, placebo-controlled, parallel-group clinical study that enrolled a sample of Italian free-living subjects with polygenic hypercholesterolemia recruited from the Lipid Clinic of the S. Orsola Malpighi University Hospital, Bologna, Italy.

Participants were required to be aged between 18 and 70 years, with moderately high levels of LDL-C (LDL-C > 115 mg/dL and< 190 mg/dL) and an estimated 10-year cardiovascular risk < 5% according to the SCORE (Systematic COronary Risk Evaluation) risk charts, not requiring lipid-lowering treatments [6]. Exclusion criteria included the following: TG < 400 mg/dL; previous history of CVD; obesity (body mass index (BMI) > 30 Kg/m^2^); diabetes mellitus; uncontrolled hypertension (i.e., systolic and diastolic blood pressures > 190/100 mmHg);a positive test for human immunodeficiency virus or hepatitis B/C/E; uncontrolled thyroid diseases; history of malignancies; use of medication or nutritional supplement that altered blood pressure levels or serum lipids (e.g., statins, ezetimibe, fibrates, omega-3 fatty acids and bile acid resins); use of anticoagulants; alcoholism; pregnancy; and breastfeeding.

**Figure 1:**
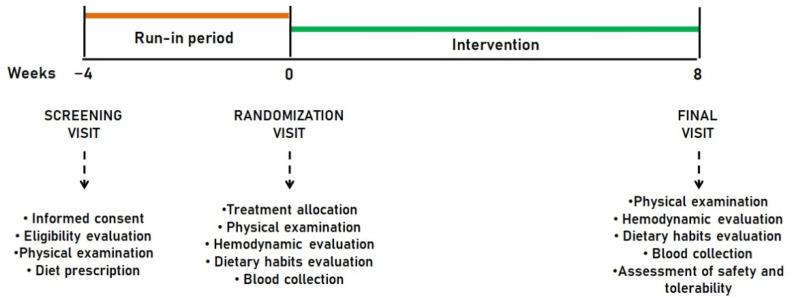
Study timeline.

Enrolled subjects were adhering to a low-fat, low-sodium Mediterranean diet for four weeks before randomization and during the study. The intervention period lasted 8 weeks. Before and after treatment, patients were evaluated for clinical status, and by the execution of a physical examination and laboratory and hemodynamic analyses. The study timeline is described in detail in Figure 1.

The study fully complied with the ethical guidelines of the Declaration of Helsinki and with The International Council for Harmonization of Technical Requirements for Registration of Pharmaceuticals for Human Use (ICH) Harmonized Tripartite Guideline for Good Clinical Practice (GCP). The study protocol was approved by the Ethical Committee of the University of Bologna and registered in ClinicalTrials.gov (accessed on 1 May 2020) (ID: NCT04574505). All patients provided written informed consent to participate.

### 2.2. Treatment

After the 1-month period of diet standardization, enrolled subjects were randomized to receive daily either an indistinguishable pill of placebo orEufortyn^®^ Colesterolo Plus containing Vazguard^®^ (caffeoylquinic acids ≥ 20.0%, total flavonoids ≥ 5.0% and Cynaropicrin ≥ 5.0%), Pycrinil^®^ (total flavanones 11.0–19.0%), artichoke (*Cynara scolymus* L.) dry extract, Ubiqsome^®^ and zinc (Table 1).

The study products were manufactured and packaged by Scharper S.p.A. (Milan, Italy), in accordance with Quality Management System ISO 9001:2008 and the European Good Manufacturing Practices (GMP), satisfying requirements in the “Code Of Federal Regulation” title 21,volume 2, part 111.

At the time of randomization, each patient was provided with boxes containing 60 tablets (either active ingredients or placebo).

Randomization was performed centrally, by computer-generated codes. Participants and investigators were blinded to the group assignment. Randomization codes were kept in a sealed envelope that was opened after study completion and data analysis.

For the entire duration of the study, patients were instructed to take a pill of the assigned treatment once daily, at about the same time each day, preferably during the evening meal.

At the end of the clinical trial, all unused pills were retrieved for inventory. Participants’ compliance was assessed by counting the number of returned pills.

### 2.3. Assessments

#### 2.3.1. Clinical Data and Anthropometric Measurements

Information gathered in the patients’ history included presence of ASCVD and other systemic diseases, allergies and medications. Validated semi-quantitative questionnaires including a Food Frequency Questionnaire (FFQ) were used to assess demographic variables, recreational physical activity and dietary and smoking habits [7].

Analysis of diet composition was performed using the MètaDieta^®^ software (INRAN/IEO 2008 revision/ADI). Data were handled in compliance with the company procedure IOA87.

Waist circumference (WC) was measured in a horizontal plane at the end of a normal expiration, at the midpoint between the inferior margin of the last rib and the superior iliac crest. Height and weight were respectively measured to the nearest 0.1 cm and 0.1 Kg, with subjects standing erect with eyes directed straight, wearing light clothes and with bare feet. BMI was calculated as body weight in kilograms, divided by height squared in meters (Kg/m^2^).

#### 2.3.2. Laboratory Analyses

Biochemical analyses were carried out on venous blood withdrawn after overnight fasting (at least 12 h). Serum was obtained by addition of disodium ethylenediaminetetraacetate (Na_2_EDTA) (1 mg/mL) and blood centrifugation at 3000 RPM for 15 min at 25 °C.

Immediately after centrifugation, trained personnel performed laboratory analyses according to standardized methods [8]. The following parameters were directly assessed: Total cholesterol (TC), triglycerides (TG), high-density lipoprotein cholesterol (HDL-C), apolipoprotein B-100 (Apo B-100), apolipoprotein AI (Apo AI), fasting plasma glucose (FPG), creatinine, high-sensitivity C-reactive protein (hs-CRP), creatine phosphokinase (CPK), gamma-glutamyl transferase (GGT), alanine transaminase (ALT) and aspartate transaminase (AST).

LDL-C was obtained by the Friedewald formula. Non-HDL cholesterol (Non-HDL-C) resulted from the difference between TC and HDL-C. The glomerular filtration rate (eGFR) was estimated by the Chronic Kidney Disease Epidemiology Collaboration (CKD-epi) equation [9].

Lipid accumulation product (LAP) was calculated as (WC − 65) × TG (expressed in mmol/L) for men and (WC − 58) × TG (expressed in mmol/L) for women [10]. Hepatic steatosis index (HSI) resulted from 8 × AST/ALT ratio + BMI (+2 for women) [11]. Finally, fatty liver index (FLI) was calculated as follows: [e^0.953×ln(TG)+0.139×BMI+0.718×ln(GGT)+0.053×WC−15.745^/(1 + e^0.953×ln(TG)+0.139×BMI+0.718×ln(GGT)+0.053×WC−15.745^)] × 100 [12].

#### 2.3.3. Blood Pressure Measurements

Blood pressure (BP) was measured in accordance with the recommendations of the International Guidelines for the management of arterial hypertension [13]. Resting systolic (SBP) and diastolic BP (DBP) were measured with a validated oscillometric device and a cuff of the appropriate size applied on the right upper arm. To improve detection accuracy, three BP readings were sequentially obtained at 1-minute intervals. The first reading was discarded, and the average between the second and the third reading was recorded as the study variable.

#### 2.3.4. Endothelial Reactivity

Endothelial function of the arterial vasculature is an important early marker of atherosclerosis, reflecting the ability of the endothelial layer to release nitric oxide (NO), modulating smooth muscle tone in the arterial wall of the conduit arteries [14].

Following the current guidelines [15], during the clinical study endothelial function was evaluated through Endocheck^®^ (BC Biomedical Laboratories Ltd., Vancouver, BC, Canada), a method embedded within the Vicorder^®^ device that guarantees very good intra- and inter-operator reliability [16]. The measurement was carried out with patients in supine position and in abstinence from cigarette smoking and caffeinated beverages for at least 12 h. After a 10-minute rest, the brachial pulse volume (PV) waveforms were recorded at baseline for 10 s and during reactive hyperemia. The BP cuff was inflated to 200 mmHg for 5 min and PV waveforms were recorded for 3 min after the cuff was released. Endothelial reactivity (ER) was calculated as change in the PV waveform area, comparing waveforms before and during hyperemia through the equation √PV2/PV1, where PV1 represents PV at the baseline and PV2 represents PV during hyperemia [17].

#### 2.3.5. Assessment of Safety and Tolerability

Safety and tolerability were evaluated through continuous monitoring during the study in order to detect any adverse event, clinical safety, laboratory findings, vital sign measurements and physical examinations. A blinded, independent expert clinical event committee was appointed by the principal investigator in order to categorize the adverse events that could possibly be experienced during the trial as not related, unlikely related, possibly related, probably related, or definitely related to the tested treatment [18].

### 2.4. Statistical Analysis

Data were analyzed using intention to treat by means of the Statistical Package for Social Science (SPSS) 25.0, version for Windows.

Sample size was calculated for the change in LDL-C. A total of 28 subjects per group were needed to detect a mean change in LDL-C at 8 weeks of 12 mg/dL with a power of 0.90 and an alpha error of 0.05. A total sample size of 60 patients (30 patients/arm) was included in the study to allow for a dropout rate of 10%.

The Kolmogorov–Smirnov test was used to test the normality distribution of the studied variables. Non-normally distribute variables were log-transformed before further statistical testing. Baseline characteristics of the population were compared using Levene’s test followed by the independent Student’s T test and by the χ^2^ test followed by Fisher’s exact test. Between-group differences were assessed by repeated-measures ANOVA followed by Tukey’s post hoc test. All data were expressed as means and related standard deviations. All tests were two-sided. A *p* level of <0.05 was considered significant for all tests.

## 3. Results

### 3.1. Efficacy Analysis

**Figure 2:**
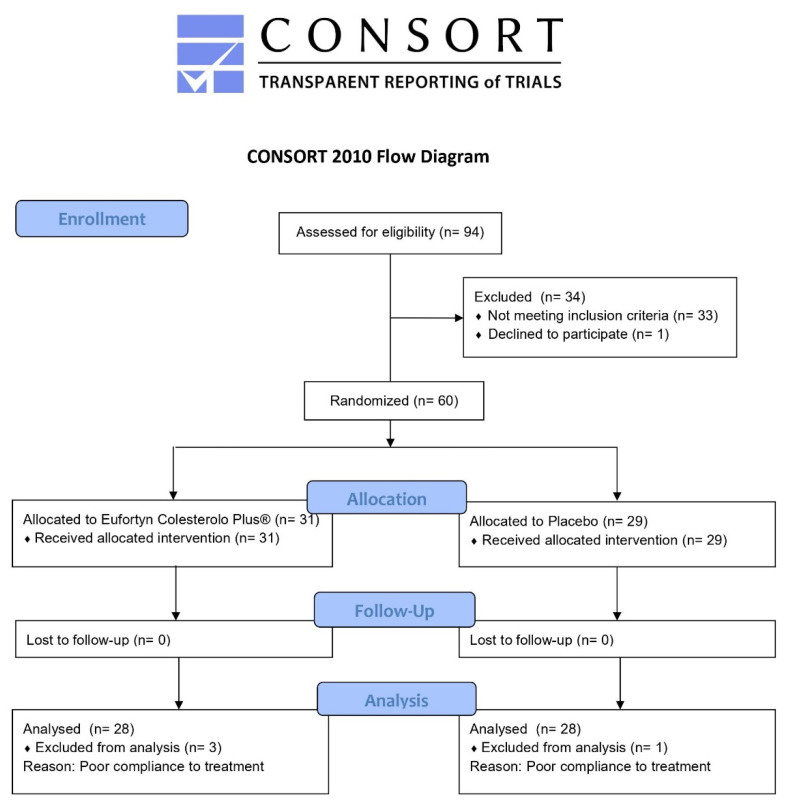
CONSORT flow diagram of the progress through the phases of the clinical study.

A total of 94 volunteers was screened, and 60 subjects underwent randomization from November 2020 through November 2021. Sixty enrolled subjects successfully completed the trial according to the study design (Figure 2).

The mean compliance to the treatment was 91 ± 2% in the active treatment group and 89 ± 2% in the placebo group. Three individuals allocated to treatment with Eufortyn^®^ Colesterolo Plus and one individual in the placebo group were excluded from analysis because of poor compliance to treatment.

The final distribution by sex did not show any significant differences between groups (*p* > 0.05), with 14 women allocated to placebo and 16 women allocated to Eufortyn^®^ Colesterolo Plus and no detectable interaction effect.

During the run-in period a non-statistically significant trend toward body weight decrease was observed in both considered groups. No statistically significant changes were recorded in the dietary habits of the enrolled individuals from randomization until the end of the study, with any changes in total energy and macronutrient intake (Table 2).

The study groups were well matched for all the considered variables at baseline, except for heart rate, which was significantly higher in the active treatment group (Table 3). At the end of the trial, dietary supplementation with Eufortyn^®^ Colesterolo Plus was associated with significant improvement in TC, LDL-C, non-HDL-C, hsCRP and ER in comparison with baseline, and with significant reductions in WC, TC, LDL-C, LDL-C/HDL-C, LAP, and FLI compared to placebo (Table 3). TC and LDL-C improved with Eufortyn^®^ Colesterolo Plus versus both baseline and placebo.

### 3.2. Safety Analysis

All participants completed the clinical trial according to the study design (dropout rate = 0%). No treatment-emergent adverse events were reported and no laboratory abnormalities occurred during the study.

## 4. Discussion

In the last decades, there has been a growing interest in the usefulness of natural compounds targeting multiple biochemical pathways [19]. In this context, dietary polyphenols have gained particular attention [20]. A growing body of evidence suggest that artichoke and bergamot standardized flavonoid extracts improve the lipid pattern in moderately hypercholesterolemic individuals, such that their use has been promoted as safe lipid-lowering agents [21,22]. Moreover, artichoke extracts and bergamot polyphenolic fraction have anti-inflammatory and antioxidant properties [23,24].

In animal models, artichoke flavonoids hinder cholesterol biosynthesis from 14-C-acetate, probably through the inhibition of 3-hydroxy-3-methylglutaryl-Coenzyme A (HMG-CoA) reductase [25]. Furthermore, artichoke flavonoids interact with liver sterol regulatory element-binding proteins (SREPBs) and acetyl-CoA C-acetyltransferase (ACAT) and increase bile acids fecal excretion [26].

In the present study, dietary supplementation with bergamot polyphenolic fraction phytosome, artichoke extracts, Q10 phytosome and zinc was effective in lowering a number of serum lipoprotein fractions, with an additional statistically significant effect on fasting plasma glucose, systemic inflammation and indexes of NAFLD. Moreover, dietary supplementation with Eufortyn^®^ Colesterolo Plus resulted in a significant improvement in ER after as early as 8 weeks. Particular attention should be paid to the prognostic meaning of this latest finding, since endothelial function is an important early marker of atherosclerosis, reflecting the ability of the endothelial layer to release nitric oxide (NO) and modulate smooth muscle tone in the arterial wall [27]. Such improvements in endothelial function have been previously found to be associated with a significant reduction in CVD risk [28].

Based on evidence from preclinical studies, artichoke’s polyphenols, such as caffeoylquinic acids, regulate the expression of a variety of genes, including vascular endothelial growth factor (VEGF), endothelin 1 (ET-1) and endothelial NO synthase (eNOS), which promote the vasodilation mediated by the endothelial cells. Moreover, luteolin andcynaroside stimulate NOS messenger ribonucleic acid (mRNA) in human endothelial cells, with consequent NO production and potential beneficial activity in the prevention of CVD [29]. Finally, coenzyme Q10 and zinc potentially have a synergistic effect with the antioxidant properties of polyphenol compounds, also improving the plasma levels of metalloenzymes (including superoxide dismutase) [30,31].

As originally reported by the CTT (Cholesterol Treatment Trialists’) meta-analyses of the statin trials, there is a linear association between LDL-C reduction and decrease in atherosclerotic CVD (ASCVD) events [32]. Robust and growing evidence highlights that this linear association is observed regardless of the LDL-C lowering approach adopted (i.e., low-fat diet, anion exchange resins, ezetimibe, etc.) [33]. In this context, the improvement in LDL-C during treatment with Eufortyn^®^ Colesterolo Plus has a clinical relevance, especially considering the concerns that have been recently raised by the European Food and Safety Agency (EFSA) regarding the safety of red yeast rice-based food supplements [34]. Moreover, unlike the other nutraceuticals specifically inhibiting cholesterol synthesis or absorption [35], bergamot polyphenolic fraction and artichoke extracts have an impact on both lipid and glucose metabolism [33,36]. For these reasons and according to our observations, Eufortyn^®^ Colesterolo Plus could have a role in the management and prevention of multifactorial metabolic disorders such as metabolic syndrome and NAFLD [37,38].

Despite the relevant findings and the practical implications, this study is not without limitations. We acknowledge the relatively small sample size, even though the study was powered for the primary outcomes and to detect between-groups differences in safety and tolerability. Moreover, the relatively short follow-up means it is not possible to assess the possible occurrence of adaptation phenomena; however, these have never been documented for polyphenols before. We also acknowledge that some investigated parameters improved in a way that was not mirrored in previous clinical studies testing bergamot polyphenolic fraction and artichoke extracts with more impressive results [39,40]. The reason might be found in the stringent eligibility criteria of the present study, which did, however, ensure high internal validity and reliability of the results. Another limitation of the study is the lack of ultrasound evaluation or transient elastography (fibroscan) of liver stiffness.

More research is needed that focuses on the underlying reasons and mechanisms of the effects observed during the study. For instance, the effect on serum lipids and inflammation is likely to be partially mediated by modification of the gut microbiota induced by polyphenols supplementation. Available experimental data suggest that supplementation with bergamot and artichoke polyphenolic fractionsis able to exert a beneficial effect on the composition of the gut microbiota [4,41]. However, to date, thereis no specific evidence related to simultaneous supplementation with these nutraceutical compounds. Finally, it is critically important that further longer-term clinical studies clarify whether the treatment-dependent changes in liver function tests reflect a significant improvement in liver stiffness.

## 5. Conclusions

In conclusion, the study shows that dietary supplementation with standardized bergamot polyphenolic fraction phytosome, artichoke extracts, Q10 phytosome and zinc safely exerts significant improvements in serum lipids, systemic inflammation, indexes of NAFLD and endothelial function in healthy subjects with moderate hypercholesterolemia.

## Figures and Tables

**Table 1:** Quantitative composition of the active treatment, namely Eufortyn^®^ Colesterolo Plus, tested in the clinical study.

Ingredients	Quantity per Tablet
Vazguard^®^ (Phytosome Bergamot Polyphenolic fraction)	1000 mg
Pycrinil^®^ artichoke d.e. (*Cynara cardunculus* L.)	100 mg
Artichoke d.e. (*Cynara scolymus* L.)	20 mg
Ubiqsome^®^ (Coenzyme Q10 phytosome)	25 mg
equivalent to Coenzyme Q10	5 mg
Zinc	5 mg

d.e. = dry extract.

**Table 2:** Diet composition (g/day) at enrollment and at the end of the clinical trial. Values are reported as mean ± SD.

Parameters	Placebo (*n*. 28)		Eufortyn^®^ Colesterolo Plus (*n*. 28)	
	Baseline	Week 8	Baseline	Week 8
Total energy (Kcal/day)	1629 ± 110	1611 ± 105	1591 ± 99	1586 ± 116
Carbohydrates (% of total energy)	54.4 ± 2.3	53.2 ± 2.5	54.5 ± 2.1	54.4 ± 2.4
Proteins (% of total energy)	18.2 ± 1.4	18.4 ± 1.6	17.8 ± 1.5	18.1 ± 1.3
Animal protein (% of total energy)	10.5 ± 0.9	9.9 ± 0.9	10.6 ± 0.7	10.9 ± 0.8
Vegetal protein (% of total energy)	7.3 ± 0.6	7.5 ± 0.8	6.7 ± 0.6	6.8 ± 0.7
Total fats (% of total energy)	27.7 ± 2.0	27.5 ± 2.3	27.2 ± 1.7	28.0 ± 2.1
Saturated fatty acids (% of total energy)	8.0 ± 0.8	8.2 ± 0.7	8.3 ± 0.7	7.8 ± 0.9
MUFA (% of total energy)	12.6 ± 1.1	12.2 ± 1.0	12.8 ± 1.0	12.3 ± 1.1
PUFA (% of total energy)	6.7 ± 0.6	6.2 ± 0.7	6.5 ± 0.5	6.7 ± 0.6
Total dietary fibers (g/day)	18.9 ± 2.5	19.1 ± 2.8	19.3 ± 2.5	18.7 ± 2.4
Cholesterol (mg/day)	191.2 ± 13.3	187.2 ± 12.3	192.8 ± 11.5	194.8 ± 10.7

MUFA = Monounsaturated fatty acids; *n* = Number of individuals; PUFA = Polyunsaturated fatty acids.

**Table 3:** Anthropometric, hemodynamic and blood chemistry parameters from the baseline to the end of the clinical trial, expressed as mean ± SD.

Parameters	Placebo (*n*. 28)		Eufortyn^®^ Colesterolo Plus (*n*. 28)	
	Baseline	Week 8	Baseline	Week 8
Age (years)	54 ± 3	-	54 ± 4	-
Body Mass Index (Kg/m^2^)	24.3 ± 3.9	24.4 ± 3.7	23.9 ± 2.9	23.7 ± 2.7
Waist Circumference (cm)	87.1 ± 14.0	87.1 ± 13.8	85.8 ± 12.4	84.8 ± 11.4 ^§^
SBP (mmHg)	135.4 ± 16.1	131.6 ± 17.7	133.8 ± 16.5	130.9 ± 17.9
DBP (mmHg)	73.5 ± 12.5	72.8 ± 10.3	73.8 ± 10.7	75.7 ± 12.8
Heart Rate (bpm)	65.5 ± 11.2	69.1 ± 12.3	73.3 ± 13.3 ^§^	71.0 ± 13.0
Total Cholesterol (mg/dL)	223.7 ± 24.7	227.7 ± 22.3	229.2 ± 20.8	214.5 ± 22.9 ^§,^*
LDL-C (mg/dL)	141.9 ± 20.1	144.9 ± 20.2	143.3 ± 17.3	131.2 ± 19.8 ^§,^*
HDL-C (mg/dL)	57.4 ± 17.6	56.5 ± 14.9	64.9 ± 18.9 ^§^	61.8 ± 18.0
Non-HDL-C (mg/dL)	166.4 ± 23.6	159.5 ± 29.0	165.3 ± 20.1	158.7 ± 23.1 *
LDL-C/HDL-C	2.7 ± 0.8	2.7 ± 0.8	2.5 ± 0.8	2.4 ± 0.8 ^§^
Triglycerides (mg/dL)	117.3 ± 70.2	126.3 ± 59.5	109.7 ± 54.3	112.9 ± 47.3 ^§^
Apolipoprotein B-100 (mg/dL)	122.0 ± 16.2	126.2 ± 17.6	118.0 ± 14.6	121.4 ± 16.9
Apolipoprotein AI (mg/dL)	154.2 ± 26.3	154.4 ± 24.2	160.4 ± 27.9	155.9 ± 28.8
FPG (mg/dL)	90.1 ± 8.5	91.4 ± 7.1	88.2 ± 11.3	89.2 ± 10.5
AST (U/L)	21.2 ± 7.2	21.2 ± 4.6	22.3 ± 4.6	22.8 ± 4.6
ALT (U/L)	22.7 ± 14.4	20.6 ± 10.3	19.2 ± 6.9 ^§^	19.0 ± 6.9
gGT (U/L)	25.2 ± 20.5	25.2 ± 17.9	21.9 ± 13.3 ^§^	22.4 ± 13.6
Lipid Accumulation Product	34.9 ± 16.4	38.1 ± 19.1	33.4 ± 16.3	32.9 ± 13.3 ^§^
Hepatic Steatosis Index	33.4 ± 5.2	32.8 ± 4.9	32.1 ± 4.1	31.7 ± 3.8
Fatty Liver Index	31.4 ± 15.9	29.5 ± 17.6	26.4 ± 14.5	26.2 ± 13.9 ^§^
CPK (U/L)	102.3 ± 74.8	108.4 ± 104.1	114.1 ± 67.8	99.2 ± 51.0
eGFR (mL/min)	85.9 ± 15.8	85.9 ± 16.1	85.6 ± 15.9	83.0 ± 16.7
hs-CRP (mg/L)	0.15 ± 0.15	0.12 ± 0.10	0.17 ± 0.24	0.13 ± 0.15 *
Endothelial reactivity	1.37 ± 0.31	1.38 ± 0.17	1.33 ± 0.27	1.43 ± 0.22 *

* *p* < 0.05 versus baseline; ^§^ *p* < 0.05 versus placebo. ALT = Alanine aminotransferase; AST = Aspartate aminotransferase; CPK = Creatine phosphokinase; DBP = Diastolic blood pressure; eGFR = Estimated glomerular filtration rate; FPG = Fasting plasma glucose; gGT = Gamma-glutamyl transferase; HDL-C = High-density lipoprotein cholesterol; hs-CRP = High sensitivity C reactive protein; LDL-C = Low-density lipoprotein cholesterol; SBP = Systolic blood pressure.

## Data Availability

Data supporting study’s findings are available from the Corresponding Author with the permission of the University of Bologna and the sponsor.
